# Supplementary material for: Adhesion of Oral Bacteria to Commercial d-PTFE Membranes: Polymer Microstructure Makes a Difference
Source: Int J Mol Sci. 2022 Mar 10;23(6):2983. doi: 10.3390/ijms23062983 (PMC8949314; doi:10.3390/ijms23062983)
Supplement: Supplementary file 1 [file ijms-23-02983-s001.zip › ijms-1622587-supplementary.pdf]

Article

Adhesion of Oral Bacteria to Commercial d-PTFE Membranes: Polymer Microstructure Makes a Difference

Gabrijela Begić [1,†], Mirna Petković Didović [2,†], Sanja Lučić Blagojević [3], Ivana Jelovica Badovinac [4,5], Jure Žigon [6], Marko Perčić [5], Olga Cvijanović Peloza [7,*] and Ivana Gobin [1]

1 Department of Microbiology and Parasitology, Faculty of Medicine, University of Rijeka, 51000 Rijeka, Croatia; gabrijela.begic@uniri.hr (G.B.); ivana.gobin@uniri.hr (I.G.)
2 Department of Medical Chemistry, Biochemistry and Clinical Chemistry, Faculty of Medicine, University of Rijeka, 51000 Rijeka, Croatia; mirnapd@medri.uniri.hr
3 Department of Surface Engineering of Polymer Materials, Faculty of Chemical Engineering and Technology, University of Zagreb, 10000 Zagreb, Croatia; slucic@fkit.hr
4 Department of Physics, University of Rijeka, 51000 Rijeka, Croatia; ijelov@phy.uniri.hr
5 Centre for Micro- and Nanosciences and Technologies & Center for Artificial Intelligence and Cyber security, University of Rijeka, 51000 Rijeka, Croatia; marko.percic@riteh.uniri.hr
6 Department of Wood Science and Technology, Biotechnical Faculty, University of Ljubljana, 1000 Ljubljana, Slovenia; Jure.zigon@bf.uni-lj.s
7 Department of Anatomy, Faculty of Medicine, University of Rijeka, 51000 Rijeka, Croatia
* Correspondence: olga.cvijanovic@uniri.hr
† The authors contributed equally to this work.

Table S1. Total surface free energies (γ^{tot}) and surface free energy components (dispersive γ^{d}; polar γ^{p}; Lifshitz-van der Waals γ^{LW}; electron pair acceptor, i.e., acidic γ^{+}; electron pair donor, i.e., basic γ^{-}) of the PTFE membranes calculated using the Owens-Wendt, Wu, and acid-base model. All values are given in mJ/m^2.

	**Owens-Wendt**			**Wu**			**acid-base**			
	γ^{d}	γ^{p}	γ^{tot}	γ^{d}	γ^{p}	γ^{tot}	γ^{LW}	γ^{+}	γ^{-}	γ^{tot}
**PTFE-W side 1**	10.69	0.01	10.7	15.01	0	15.01	10.33	0	0	10.33
**PTFE-W side 2**	10.08	0.05	10.13	14.49	0	14.49	9.7	0	0.33	9.7
**PTFE-B side 1**	8.5	0	8.5	13.49	0	13.49	8.49	0	0	8.49
**PTFE-B side 2**	11.95	0	11.95	17.4	0	17.4	13.41	0	0.52	13.41
